# Synopsis of the Discussions of the American Dental Society of Europe

**Published:** 1874-11

**Authors:** C. M. Wright

**Affiliations:** Sec., Geneva, Switz.


### A SYNOPSIS OF THE DISCUSSIONS OF THE AMERICAN DENTAL SOCIETY OF EUROPE.

*Geneva, Switz., Hotel de la Paix, July 2d*, 1874.

Pivot teeth: Essay by Dr. Van Marter, in which a method of pivoting teeth is described and advocated. Uses plain plate teeth with thin lining of platinum. The lining is left long enough to be burnished down over the face of the root. A hole is cut through this elongated lining, corresponding with the hole in the root, through which a long platinum pivot is passed, and, after fastening with wax and removing from the mouth, is invested in sand and plaster and soldered with fine gold. The pivot is then fastened into the root with gutta percha or shellac, and the joint is perfectly tight. He tried this method for six years with great success.

Dr. Field asked how it was possible to conserve a root of a tooth with a metal cap and gutta percha when so many of our best fillings do not form the perfect joint claimed, and these fillings are made in plain view of the operator and not under a cap of metal. Would prefer a gold crown of adhesive gold to be seen at the neck of the tooth.

Is not opposed to pivoting and would not place his theory against six years experience.

Dr. Terry considers the method described by the essayist superior to the more difficult and more expensive one of Dr. Field; considers the operation as secure and as sure of conserving the root as the one first referred to. Has practiced this method of pivoting for several years and prefers it to any other he has knowledge of.

Dr. Kingsley: The operation of pivoting is not a favorite one with him, but would ask if the method of burnishing down a thin piece of platinum will make a better joint than the old method of grinding down a pivot tooth, by a careful operator and then applying his gutta percha or shellac or any other substance to secure the metal or wooden pivot? Considers the making of a complete cap over the exposed face of the root with cohesive gold the only method of perfectly excluding moisture.

Dr. Terry regards this method as having particular advantages over the old on account of the better articulation obtainable with the plain plate tooth. Has used this method even in bicuspid cases with success.

Dr. Wasson gives preference to the old fasioned wooden pivot and though has seen cases made by amateur dentists that have done good service for 15 to 20 years, never promises much in regard to durability of pivot teeth.

Subject: Local Peculiarities of Diseases of the Teeth of Europe.

Short paper by C. M. Wright.

Dr. Abbott stated that in Berlin generally teeth lacked phosphate of lime. The soil lacked it and even hen's eggs are soft. In Norway and Sweden the teeth are denser and less liable to the diseases. Thinks the teeth in England and America are generally better, denser, harder and have more resisting power to disease than the teeth in Europe generally. In Berlin soft chalky teeth were almost the rule.

Dr. Gregory has seen more cases of loosening and loss of teeth from the accumulation of tartar in Lyons than in any part of the world he has observed. Inflammation and suppuration of the gums with a catarrhal odor is often found here, which he thinks is augmented or caused by the excessive use of chloral as a dentrifice. Has strongly opposed its use in Lyons.

Dr. Abbott considers charcoal as rather a preventive for inflammation and spongy conditions of the gums. Has observed often that the gums of persons who use charcoal as a dentrifice, present a tattooed appearance but are free from inflammation and sponginess.

Dr. Field finds the teeth of the people of Geneva are soft. Formerly cut away too much in the preparation of the cavities owing to general softness of the the tooth substance. Experience has taught him not to cut so freely. Soft spongy gums prevail. Considers this a result of a want of exercise with the brush and recommends brushing the gums hard (this opinion was generally objected to.) Abrasion of the alveola and recession of the gums are quite as common when the teeth are soft and the gums spongy as where a harder quality of tooth is found.

Dr. Terry: In Zurich the teeth of the laboring classes are worse than those of the upper and wealthier class. Considers this a result of the mode of living, bread and wine being the staple article of diet and the wine being not of the rich quality that the upper class use.

Dr. Kingsly: If there is any noticeable peculiarity of the teeth or mouths of Russians, French or Germans that he had observed it is as Dr. Gregory said, the spongy, inflammatory gums from uncleanliness. Does not think friction of the gums tends to hardening of them, friction should be applied to the teeth and *not* the gums (issue taken by Dr. Field.) Insists that as the diseased conditions are mostly found in the buccal and labial surfaces, only want of cleanliness is the cause. Hard brushing of the gums is not only unnecessary for cleanliness, but positively injurious In these cases of sponginess simple cleanliness of the teeth for a time will cure them.

Dr. Paetsch does not believe in any peculiarities of different nations; finds the same varieties in American, Russian, French and German teeth, and a healthy person of one nationality will present the same teeth as another of a different nationality.

Dr. Dumont agrees with the last speaker, temperament, diathesis and not nationality affect the teeth. Scrofulous teeth are scrofulous teeth all over the world.

Dr. Wasson thinks from Louisiana and other western States of America, that nationality has severe influence on the diseases of the teeth even in different localities. In England and France found gum diseases and defective teeth. In Egypt and Palistine found the teeth not so predisposed to caries nor are the inhabitants troubled with tooth ache as in most other countries.

The Romans, as a rule, have comparatively good teeth. The water of Rome is strongly impregnated with lime, and caculus abounds—finds chemical abrasions and diseases of the gums.

Dr. Spear: In the mountain districts of Berne, bread, milk and cheese are the staples of diet among the peasants. Has never found tartar deposit on their teeth, and the teeth are unusually good. In the valley, on the contrary, the teeth are unusually bad, and tartar is found in quantities. Thinks geological reasons might be offered for some of the diseases of the mouth and teeth.

Dr. Van Marter mentioned that the teeth of the numerous children engaged in the chocolate factories of Neuchatel are invariably affected with caries.

The next subject, "What does experience teach to be the best material for filling the teeth," elicited the usual discussion.

Essay read by C. M. Wright.

Dr. Abbot has never used amalgam, but has favored Abbey's soft foil, in certain cases of very soft, chalky teeth. Has for a period of twelve years employed occasionally gold and tin foil together in the following manner: a sheet of gold foil is laid upon a sheet of tin-foil, and cut at proper widths for the cavity, then rolled between the finger and thumb into a loose rope, the tin being outside. This is introduced as any rope filling, not requiring so hard pressure as gold alone, yet making a harder filling than tin alone. After this filling has been in a few months, the surface is found oxydized like an amalgam, and would often be taken for amalgam, even upon cutting into it; but the walls, sides and bottoms of the cavity, even after years, have remained very clear and free from further attacks of caries. The combination of these metals, from the electrical or other effects, have proved in these very soft teeth, a very valuable agent for their preservation. [Great surprise was expressed by the society at this novel method]. I do not claim this as my method, but a friend [the reporter did not get the name] recommended me to try this method twelve years ago, and practice has proved it a very valuable one to me—hoped the society would try it, no matter what scruples or theories they may entertain, and report on it next year. Several dentists in Berlin are usuing it in these peculiar cases.

These methods were discussed by the society, and the opinion prevailed that the injurious electrical effects would condemn it; but as Dr. Abbot has had twelve years experience, and has carefully noted the results, the discussion was really *theory versus facts.*

Dr. Terry thought amalgam never had a fair trial with other fillings; for various reasons, the first class dentists had not used it, and many others only in the most unfavorable cases. He, in common with every other member of the society, placed his reliance upon gold.

Dr. Dumont stated that when Dr. Abbot and himself went to Berlin, twenty-three years ago, they found only amalgam dentists. No one used gold, and they had resolved not to use it at all, but to discourage it in every way, and encourage a higher standard of dentistry by employing the precious metal, gold.

Dr Wright: We have here the moral effects of gold, it raises the standard of our operations. There is also an economical effect—it puts gold into our pockets. These are two very powerful and important reasons for the employment of gold in filling teeth. They are reasons that every man, and especially he who is making a reputation in a foreign country, must consider. They are private reasons, and reasons of policy, but not really scientific reasons for the employment of any material.

Dr. Abbot has not *strong* scientific reasons againts a discriminating use of amalgam—has never from this principle or policy used it himself. Has, however, prepared cavities, and in rare cases permitted amalgam to be introduced by others.

Other subjects discussed by society, but as they will appear again next year for discussion no report was made.

C. M. WRIGHT, Sec.

